# Nanoinformatics: spanning scales, systems and solutions

**DOI:** 10.3762/bjnano.17.28

**Published:** 2026-03-05

**Authors:** Iseult Lynch, Diego S T Martinez, Kunal Roy, Georgia Melagraki

**Affiliations:** 1 School of Geography, Earth and Environmental Sciences, University of Birmingham, Edgbaston, B15 2TT Birmingham, United Kingdom; 2 Brazilian Nanotechnology National Laboratory (LNNano), Brazilian Center for Research in Energy and Materials (CNPEM), Campinas, Sao Paulo, Brazil; 3 Drug Theoretics and Cheminformatics (DTC) Laboratory, Department of Pharmaceutical Technology, Jadavpur University, Kolkata 700032, India; 4 Division of Physical Sciences and Applications, Hellenic Military Academy, Vari 16672, Greece

**Keywords:** artificial intelligence, in silico approaches, machine learning, nanoinformatics, nanomaterials functionality, nanotoxicity, sustainability

Nanoinformatics (as an offshoot of chemoinformatics) refers to the combination of physical chemistry and materials theory with in silico approaches to address key questions including the prediction of (nano)materials (NM) functionality, nanomaterials fate in the environment, toxicity or therapeutic ability, and recyclability. As the properties of nanomaterials themselves span several scales, from electronic, atomistic, mesoscopic to continuum, and are highly dynamic and context dependent (i.e., interact with and are transformed by their surroundings as well as impacting on their surroundings), they introduce new challenges for naming, describing, and representing them, and require the combination of physics-based and data-driven modelling approaches. The emergence of artificial intelligence and machine learning approaches, both causal and generative, are opening up new opportunities for exploring the materials and chemical space to develop new as yet undiscovered nanomaterials, for optimising in parallel the functionality, safety and sustainability of nanoscale and advanced materials (so-called multi-criteria optimisation), and as a key driver of the knowledge and digital transitions that will underpin the next decade of industrial innovation as shown schematically in Figure 1.

**Figure 1:**
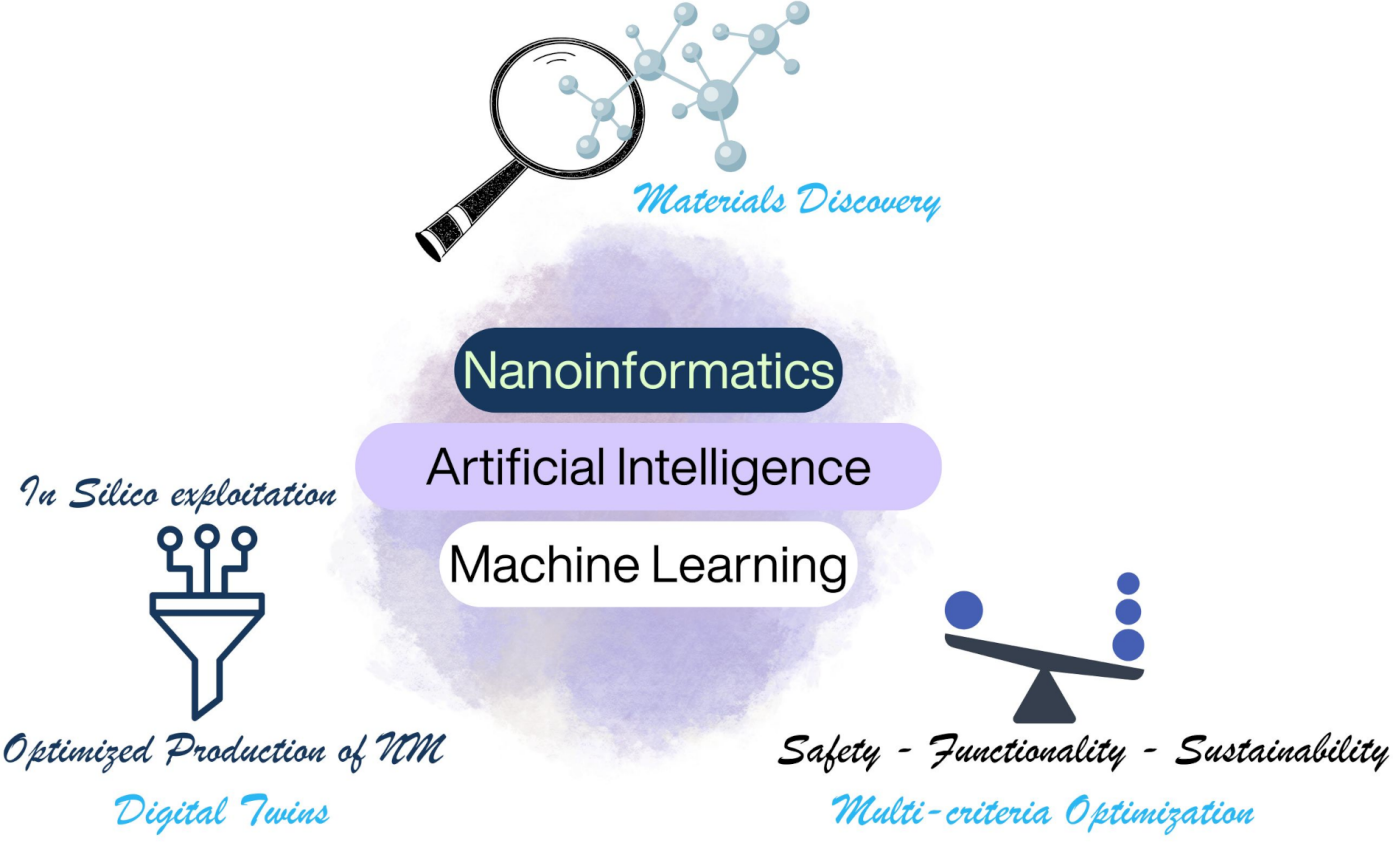
Schematic illustration of the central role of nanoinformatics in driving materials discovery, multi-criteria optimisation of NMs safety, sustainability and functionality, and use of digital twins for in silico design and testing of nanomaterials prior to production. Illustration of Interconnected Molecules Symbolizing Chemical Bonds ©vectormine via https://www.Canva.com; Filter Lineal Icon Set @premystd via https://www.Canva.com; Balance, Scale Icon @shaharea via https://www.Canva.com; Watercolor stain @benedak via https://www.Canva.com; Magnifying Glass Illustration @joanna-rzonca via https://www.Canva.com. The content depicted in Figure 1 is not subject to CC BY 4.0.

This thematic issue includes 13 articles (10 original research papers, two perspectives, and one review paper) that provide a snapshot of recent exciting developments in nanoinformatics, and is an output from the Beilstein Nanotechnology symposium [1] of the same name, held in October 2022. The advances presented are clustered around four key needs, including (i) prediction of nanomaterials physicochemical properties, structural features, and biomolecule interactions using both physics-based and machine-learning (ML) approaches; (ii) prediction of nanomaterials toxicity including development of novel toxicity-related descriptors; (iii) solution-focussed approaches applying advanced artificial intelligence (AI) and ML approaches to nanomaterials disease therapy, environmental remediation, and to support implementation of the framework for Safe and Sustainable by Design (SSbD); and (iv) infrastructure and tools to underpin the implementation of nanoinformatics.

Given the heterogeneity of properties of nanomaterials reported in experimental papers, the ability to predict or impute physicochemical properties as inputs for quantitative structure/activity/property relationship (QSAR/QSPR) models is critical. Moncho et al. surveyed the nanomaterials QSAR literature to determine the variety of calculated and experimental features used to define and describe nanomaterials, and proposed a classification of the descriptors into those that directly describe a component of the nanoform (core, surface, or structure) and those that indirectly reflect its structure (experimental features related to the nanomaterial’s behaviour, preparation, or test conditions) [2]. Voyiatzis et al*.* computationally studied, using atomistic molecular dynamics simulations, the morphological transformations (from molten/amorphous to crystalline) during rapid cooling of 1–8 nm spherical gold and platinum nanoparticles (NPs), which are challenging to experimentally measure. Using computational descriptors commonly used in nano-QSAR models, such as the potential energy of surface atoms and the water–NP surface energy, the model predicts that Pt NPs are more toxic than Au NPs, based on their surface properties, which drive reactivity [3]. Amini et al. combined atomistic molecular dynamics, a coarse-grained model of protein adsorption, and kinetic Monte Carlo simulations to predict the protein corona composition formed on aluminium surfaces with different crystal faces, (i.e., (100), (110), and (111)) from a simplified model of milk consisting of the six most abundant milk proteins found in natural cow milk and lactose, the most abundant sugar found in dairy products, based on their corresponding interaction strengths. The resulting freely accessible multiscale computational model enables predictions of the binding strength, preferred orientations, and relative abundance of the specified molecules on the specified material surfaces giving an insight into the mechanisms of bio–nano interaction [4]. Varsou et al. demonstrated a novel approach to evaluate the performance of different models for the same endpoint (zeta potential of nanomaterials) trained using a common dataset, through the generation of a consensus model, leading to increased confidence in the overall model predictions and underlying models. The consensus models outperform the individual models (*k*NN/read-across, random forest regression, AdaBoost regression, Stacked PLS – quantitative read-across structure–property relationship (q-RASPR), and Stacked MLP – q-RASPR), resulting in more reliable predictions overall, and suggesting that this approach could enhance regulatory acceptance of in silico new approach methodologies for hazard and risk assessment of nanomaterials [5].

A major topic in the field is to identify the drivers of nanomaterials toxicity, through understanding which physicochemical properties or atomistic properties are most strongly correlated with – and thus predictive of – toxicity, often measured in vitro as cytotoxicity. To address this question, Roy and Roy constructed a quantitative structure–property relationship (QSPR) model with 132 metal oxide (MeO*_x_*) nanomaterials to understand the possible mechanisms of cell membrane damage and the role of zeta potential (a proxy for surface charge) in particular. The results showed that zeta potential, along with periodic-table-based descriptors such as an increase in oxygen count, electronegativity, and formation of a cationic charge, all influence cell membrane damage, and had the potential to influence oxidative damage through free radical accumulation, which could lead to changes in the survival rate of cancerous cells, also offering insights for potential nano-based cancer therapeutics [6]. Focusing on one specific MeO*_x_* nanomaterial, nano-TiO_2_, and a kidney epithelial cell – human renal cortex proximal tubule epithelial (HK-2) – Roy and Roy explored the potential for the nano-TiO_2_ to act as a carrier for other heavy metals such as Cd, Zn, Pb, Co or Ni into the cells – a so-called Trojan Horse mechanism. Using an ensemble learning approach that implements gradient boosting and bagging algorithms, four models were developed (i.e., a random forest, AdaBoost, Gradient Boost, and Extreme Gradient Boost) and used to establish statistically significant relationships between the structural properties of the TiO_2_ nanomaterials and the cause of cytotoxicity. The experiment-independent periodic table descriptors utilised here were found to produce better predictions than quantum chemical descriptors in previous studies, demonstrating the power of ML in conjunction with periodic table descriptors to predict co-exposure effects [7]. Further extending the concept of experiment-independent periodic table descriptors, Kar and Yang introduced 3rd-generation periodic table descriptors (i.e., atomic radius, crystal ionic radii, density of the metal, electron affinity, and ionization energy) which complement and extend the seven first- and sixteen second-generation periodic table descriptors, as a means to model the toxicity of MeO*_x_* nanomaterials to zebrafish embryo – measured as impacts on the enzymatic activity of the hatching enzyme ZHE1. The developed nano-quantitative read across structure–toxicity relationship (nano-qRASTR) model, featuring three attributes, outperformed the previously reported simple QSTR model, and enabled prediction of zebrafish embryo toxicity of 35 diverse MeO*_x_* nanomaterials, thus helping to fill the current gap in the toxicity data for zebrafish [8].

A major driver of the development of nanomaterials, nanoinformatics and ML/AI is the potential for solutions to real-world issues, whether in nanomedicine, nano-enabled agriculture, or environmental remediation. Improving the efficacy of targeted therapies and minimizing off-target effects are key challenges in nanomedicine. To address these, Dasgupta et al. mapped the structural fingerprints of ligands governing the cellular uptake of MeO*_x_* nanomaterials based on classification-based ML models (i.e., Bayesian classification, random forest, support vector classifier, and linear discriminant analysis) applied to multiple cell types (pancreatic cancer cells (PaCa2), human endothelial cells (HUVEC) and human macrophage cells (U937)). The best model for each cell type was identified, and the structural fingerprints/features governing the cellular uptake were analysed as a basis for programming higher cellular uptake efficiency and better therapeutic response [9]. He et al. used additive AI-based approaches to identify nanoparticle systems for delivering drugs to treat neurodegenerative diseases. Their method overcomes two major challenges: the scarcity of data on nanomaterial-based neural drug delivery and the enormous number of possible nanomaterial–drug combinations. The approach combines information fusion, perturbation theory, and machine learning to create a unified dataset comprising 4403 neuronal drug delivery assays from ChEMBL and 260 nanoparticle cytotoxicity assays from journal articles on which linear discriminant analysis and artificial neural network algorithms were applied. The resulting models were effective as an initial rapid pre-screening of putative nanoparticle-based drug delivery systems to treat neurodegenerative disease [10]. Moving into the realm of mixture toxicity and environmental impacts of nanomaterials, Petry et al. investigated the interaction of graphene oxide (GO) with tannic acid (TA) and its consequences for GO toxicity to the earthworm *Caenorhabditis elegans*. Reactive classical molecular dynamics and ab initio calculations revealed that TA preferentially binds to the most reactive sites on GO surfaces via oxygen-containing groups or the carbon matrix. The binding energy was dominated by van der Waals interaction forces. A dose-dependent mitigating effect of TA on the toxicity of GO was observed, and attributed to the surface interactions between TA and GO as well as to the inherent biological properties of TA in *C. elegans*. The findings provide insights that can be utilised for the design of safer nanomaterials, as part of the Safe and Sustainable by Design (SSbD) framework [11]. Finally, providing a forward-looking perspective, Melagraki discussed the transformative potential of ML and AI when applied to the design of safer and more sustainable nano- and advanced materials. The ability to computationally screen candidate materials before ever producing them and the concept of digital twins – of nanomaterials, of their production lines, their interaction partners, or even of the environmental compartments into which they may be released – enable both industrial and regulatory innovations in a safe space. However, it requires a strong focus on overcoming barriers such as the perception of models as black boxes through, for example, explainable AI [12].

The final group of papers explores some of the underpinning services and technologies needed to enable nanoinformatics, including data management workflows to combine, harmonise, and organise datasets in machine-actionable formats. Le Piane et al. explored the commonalities among advanced digital technologies, such as high-performance computing, AI/ML and data management workflows. Using a digital, data-centric methodology, the proposed approach to integrating methodologies utilises structured information management approaches to establish a framework for representing materials-related information and facilitate interoperability across diverse tools. The approach highlights the role of digital twins in nanomaterials development and examines the impact of knowledge engineering in establishing data and information standards to facilitate interoperability [13]. Punz et al. presented a practical approach to capturing both nanomaterials and data provenance, via the InstanceMaps tool, which allows users to document research workflows of increasing complexity, including documentation of: (i) synthesis, functionalisation, and characterisation of nanomaterials; (ii) assays used to assess the transformations of nanomaterials in complex media; and (iii) assays used for the assessment of the toxicity of the nanomaterials, for example using standardised *Daphnia magna* assays or human immunotoxicity assessment using cell lines and primary cellular models. Another example demonstrated the use of the instance map approach for the coordination of materials and data flows in complex multi-partner collaborative projects, providing information on both materials and data flows in a user-friendly approach to metadata capture [14].

As this snapshot shows, nanoinformatics is an exciting and fast moving area with much to look forward to in terms of nanoinformatics enabled innovations, integrations, and impacts.

Iseult Lynch, Diego S. T. Martinez, Kunal Roy, and Georgia Melagraki

Birmingham, Campinas, Kolkata, and Vari, October 2025.

## Data Availability

Data sharing is not applicable as no new data was generated or analyzed in this study.
